# A nanostructured solid-contact electrode for real-time monitoring of copper nanoparticle dynamics and environmental analysis

**DOI:** 10.1186/s13065-025-01692-y

**Published:** 2025-12-16

**Authors:** Sherif M. Eid, Menna S. Elsherbiny, Mahmoud M. Abbas, Maha F. Abdel Ghany, Khadiga M. Kelani

**Affiliations:** 1 Analytical Chemistry, Faculty of Pharmacy, October 6 University, 6 October City, Giza, Egypt; 2 Analytical Chemistry Department, Faculty of Pharmacy, Modern University for Technology and Information, Cairo, Egypt; 3 Pharmaceutical Analytical Chemistry Department, Faculty of Pharmacy, Ain Shams University, Abbassia, Cairo, 11566 Egypt; 4 Department of Medicinal Chemistry, Faculty of Pharmacy, Galala University, New Galala City, Suez, 43713 Egypt; 5 Analytical Chemistry Department, Faculty of Pharmacy, Cairo University, El-Kasr El-Aini Street, Cairo, 11562 Egypt

**Keywords:** Copper ion-selective electrode, Solid-contact electrode, Polyaniline nanoparticles, β-cyclodextrin, Real-time monitoring, Nanoparticle synthesis, Environmental water analysis

## Abstract

Copper ions (Cu²⁺) are environmentally significant due to their toxicity and widespread industrial use. Traditional detection methods are often time-consuming and not amenable to real-time analysis. This study presents the development of a solid-contact ion-selective electrode (SC-ISE) that enables real-time potentiometric monitoring of Cu²⁺ ions during copper nanoparticle (CuNP) synthesis, degradation, and environmental analysis. A novel SC-ISE was fabricated by modifying a screen-printed electrode with polyaniline nanoparticles as the ion-to-electron transducer, and a PVC membrane doped with β-cyclodextrin and Reinecke’s salt for Cu²⁺ selectivity. The sensor’s electrochemical performance was characterized under various conditions and applied for kinetic and thermodynamic studies of CuNP synthesis using L-ascorbic acid. The electrode exhibited a near-Nernstian slope (28.05 mV/decade) across a wide concentration range (10⁻⁸ to 10⁻² mol L⁻¹), a detection limit of 7.5 × 10⁻⁹ mol L⁻¹, fast response time (≤ 10 s), and stable performance within pH 6–9. Real-time monitoring of CuNP formation enabled calculation of activation energy (Ea = 34.06 kJ mol⁻¹), enthalpy (ΔH = 31.21 kJ mol⁻¹), and Gibbs free energy (ΔG > 77.9 kJ mol⁻¹), confirming an endothermic, non-spontaneous process. The electrode also tracked Cu²⁺ release during oxidative nanoparticle degradation and quantified Cu²⁺ in spiked environmental waters with 95.8–98.9% recovery. This study demonstrates, for the first time, the use of a Cu²⁺-selective SC-ISE as a portable kinetic and thermodynamic probe for nanoparticle processes, while also providing a green, low-cost platform for environmental water monitoring.

**Supplementary Information:**

The online version contains supplementary material available at 10.1186/s13065-025-01692-y.

## Introduction

Copper is an essential trace element naturally present in soil, water, and biological systems, where it plays key roles in enzymatic and metabolic processes. However, excessive exposure to copper salts poses significant health risks, disrupting enzyme activity and contributing to diseases such as rheumatoid arthritis, melanoma, and sclerosis [1, 2] Neurological disorders including Menkes disease, Wilson’s disease, and Alzheimer’s disease have also been linked to abnormal copper metabolism. Beyond its biological relevance, copper contamination in water sources remains a critical environmental issue due to the widespread industrial and agricultural use of copper compounds [3]. 

In recent years, copper has also been utilized in the form of nanoparticles (CuNPs) [4]. which exhibit unique antimicrobial properties and find application in wastewater treatment [5], pharmaceuticals, cosmetics, wound healing, and as additives in coatings, textiles, and paints [6]. The growing incorporation of CuNPs into consumer products has heightened concerns regarding their environmental release, potential toxicity, and long-term stability. As such, the accurate detection and monitoring of copper ions and nanoparticles is increasingly important for both environmental protection and public health.

Several analytical techniques have been employed for copper quantification, including spectrophotometry, including spectrophotometry [7, 8], chromatography [9, 10], voltammetry [11, 12] and potentiometry [13, 14]. While these methods provide sensitivity and selectivity, most require sophisticated instrumentation, trained personnel, and are not suitable for continuous or on-site monitoring. In contrast, potentiometric ion-selective electrodes (ISEs) offer a simple, inexpensive, and rapid alternative that enables direct detection of ionic species [15–17]. Depending on their construction, ISEs may be classified as glass, liquid-based, or solid-state electrodes [18]. Although attractive for practical use, conventional ISEs often suffer from limited selectivity and potential drift, which can compromise accuracy in complex matrices [19]. 

Recent advances in miniaturized screen-printed electrodes (SPEs) have significantly improved the portability and performance of potentiometric devices [20, 21] When combined with nanomaterials, SPEs exhibit enhanced selectivity, sensitivity, and stability [22, 23]. Different modification strategies such as ink mixing, drop casting, and electrodeposition allow the incorporation of metallic nanoparticles or functional polymers, producing tailored surfaces for targeted analytes [24]. Conducting polymers such as polyaniline (PANI) are especially promising as ion-to-electron transducers, improving electrode stability and response reproducibility.

Several previous studies have reported Cu²⁺-selective electrodes for environmental or analytical applications, including natural water, soil, and industrial samples. For instance, De Marco et al., discussed the use of ion-selective electrodes for Cu²⁺ determination in diverse environmental matrices such as natural waters and soils [25]. Subsequent works have demonstrated analytical improvements using nanocomposite and solid-contact architectures, such as CuO-MWCNT [26], ionic liquid carbon nanotube nanocomposite [27], and hydrophobic NiCo₂S₄/PFOA interfaces [28]. Other investigations extended these sensors to microfluidic formats [29] or environmental monitoring in sediment pore waters [30], sediment seawater interface [31] and other environemntal samples [32, 33].

While these studies primarily focused on Cu²⁺ quantification in static aqueous samples, none have applied Cu²⁺-selective electrodes to monitor dynamic redox transformations, such as the synthesis and degradation of metallic copper nanoparticles in real time. The present work introduces this new concept by employing a β-cyclodextrin/PVC-based Cu²⁺-selective membrane integrated with a polyaniline solid-contact layer, capable of continuously tracking Cu²⁺ concentration changes during nanoparticle formation and subsequent oxidative dissolution.

This approach transforms the classical ion-selective electrode from a static concentration sensor into a kinetic monitoring tool, enabling extraction of thermodynamic (ΔH, ΔG) and kinetic (Ea) parameters directly from potentiometric data, an aspect absent in all previously published Cu²⁺ ISE studies. Therefore, to the best of our knowledge, this work represents the first application of a potentiometric Cu²⁺ sensor for real-time monitoring of nanoparticle synthesis and degradation, combining electrochemical selectivity with kinetic insight.

Furthermore, the sensor was validated in environmental water samples, demonstrating its applicability for practical monitoring. This dual role, as both an analytical tool for nanoparticle process tracking and an environmentally relevant sensor, highlights the novelty and significance of our approach.

## Experimental

### Devices

A JENWAY device model 3505 pH/mV/Temperature Meter (Dunmow, England) coupled to saturated Calomel Hg/HgCl_2_ reference electrode (Beckman, USA) was used for all potentiometric and pH measurements. A three-millimeter single desk graphitic carbon disposable screen-printed electrode (Zensor R&D^®^, Taichung city, Taiwan) was utilized. For nanoparticle formation and characterization, a heating magnetic stirrer (VELP Scientific Srl, Velate MB, Italy), a Shimadzu UV dual beam spectrophotometer (Model 1650, Japan) connected to UVprobe software, and a Transmission electron microscope (TEM), (JEOL USA, INC., Model JEM-1230) were used.

### Chemicals

Ammonium tetra(thiocyanato)–diammine chromate (III) known as Reinecke’s salt (NH_4_[Cr(NCS)_4_(NH_3_)_2_].H_2_O), Bis(2-Ethylhexyl) phthalate (DOP) (Log *P* = 8.025), tetrahydrofuran (THF), polyvinyl chloride (PVC) (Log *P* = 0.593), and ascorbic acid were purchased from Sigma-Aldrich, USA. Copper (II) sulfate pentahydrate (CuSO_4_.5H_2_O) and Beta-Cyclodextrin (β-CD) (Log *P* = − 12.396) were obtained from Organik Kimya Co., Turkey. Pure deionized water was used.

### Standard solutions

An accurately weighed amount of 0.06242 g of CuSO₄·5 H₂O was dissolved in phosphate buffer (pH 7.0) and diluted to 25 mL in a volumetric flask to prepare a 1 × 10⁻² mol L⁻¹ stock solution. A series of six tenfold serial dilutions was then performed to obtain standard solutions ranging from 1 × 10⁻³ to 1 × 10⁻⁸ mol L⁻¹. Specifically, 2.5 mL of each higher concentration solution was transferred to a 25 mL volumetric flask and diluted to volume with phosphate buffer to prepare the next lower concentration. This stepwise dilution process ensured consistent and accurate preparation of all working solutions.

The phosphate buffer (pH 7.0) was prepared according to the United States Pharmacopeia (USP) method by dissolving an appropriate amount of monobasic potassium phosphate (KH₂PO₄) in distilled water, followed by the gradual addition of 0.2 mol L⁻¹ sodium hydroxide (NaOH) solution while continuously monitoring the pH. The pH was adjusted to exactly 7.0, and the solution was then diluted to the desired final volume with distilled water [34]. 

### Fabrication and optimization of copper-selective sensor

#### Preparation steps of polyaniline nanoparticles

Polyaniline (PANI) nanoparticles were prepared according to the method described by Moulton et al. [35]. with slight modifications. In summary, the chemical polymerization was conducted in a water bath maintained at 20 °C. Equimolar concentrations (1.3 mol L⁻¹) of aniline and sodium dodecyl sulfate (SDS) were dissolved in 100 mL of distilled water within a round-bottom flask sealed with a stopper. The mixture was subjected to mechanical stirring for 1 h to ensure thorough homogenization. Subsequently, 100 mL of ammonium persulfate solution (1.3 mol L⁻¹) was added to the resulting milky aniline/SDS emulsion to initiate polymerization. The reaction proceeded for 2.5 h, yielding a dark green dispersion of PANI. The polymerized product was dialyzed against deionized water for 48 h using a dialysis membrane with a molecular weight cutoff of 12,000 Da to remove unreacted species. Post-dialysis, the dispersion was centrifuged at 10,000 rpm for 10 min, followed by four successive washes with deionized water to eliminate residual SDS. Finally, the obtained PANI nanoparticles were suspended in xylene (10% w/w), stored in an amber glass bottle with a sealed cap, and kept protected from light.

#### Copper selective membrane fabrication procedures

In a petri dish, 0.01 g Reinecke’s salt (ionophore) was accurately weighed and combined with 0.02 gm of Beta-Cyclodextrin, a hydrophobic material to improve mechanical and thermal stability. Additionally, 0.02 gm of PVC was added as the base for the polymeric membrane. These ingredients were thoroughly blended using 0.35 mL of dioctyl phthalate (DOP). Approximately 9.5 mL of tetrahydrofuran (THF) was required to dissolve the solid ingredients.

Using a micropipette, 10 µL of the synthesized PANI nanoparticle suspension was precisely applied to the surface of the SC-ISE disc, forming a uniform dry film. Subsequently, 20 µL of the prepared membrane blend was deposited onto the electrode via drop-casting and allowed to air-dry for 4 h. To improve the membrane’s selectivity toward copper ions, the coated electrode was immersed in a 1 × 10⁻² mol L⁻¹ copper (II) stock solution for conditioning for a period of 24 h.

#### Influence of pH on the electrochemical response of fabricated sensor

To investigate the impact of pH on the sensor’s electrochemical performance, two copper ion concentrations 1 × 10⁻⁴ mol L⁻¹ and 1 × 10⁻⁵ mol L⁻¹ were employed. The sensor potential was measured over the whole pH range of 1.5 to 12. pH adjustments were carried out by the incremental addition of 0.1 mol L⁻¹ HCl or 0.1 mol L⁻¹ NaOH, as required. This procedure facilitated the identification of the pH range that yields the most stable and optimal sensor response.

#### Influence of temperature on the performance of the fabricated sensor

The influence of temperature on the operational performance and stability of the Cu²⁺-selective sensor was assessed by analyzing copper sulfate calibration solutions with concentrations ranging from 1 × 10⁻⁸ to 1 × 10⁻² mol L⁻¹. These solutions were subjected to controlled thermal conditions at 60, 70, and 80 °C. For each temperature setting, the electromotive force (EMF) was recorded corresponding to each concentration, and the resulting data were used to evaluate and derive the associated calibration parameters.

#### Effect of stability time on the stability of the fabricated sensor

To assess the sensor’s dynamic response characteristics and ensure accurate data acquisition within a constrained time window, the response time was evaluated by monitoring the stabilization period following a tenfold stepwise increase in copper sulfate concentration. A stopwatch was used to record the time required for the Cu²⁺-selective SC-ISE to reach a stable potential, defined as within ± 1 mV of the final equilibrium value, for each concentration transition.

#### Calibration curve construction

The calibration of the copper-selective sensor was conducted by sequential dilution of a 1 × 10⁻² mol L⁻¹ copper sulfate solution, while continuously monitoring the changes in electromotive force (EMF). A calibration curve was constructed by plotting the recorded potential values against the logarithm of the corresponding copper ion concentrations. All EMF measurements were carried out using a double-junction Hg/HgCl₂ calomel reference electrode, ensuring that the membrane potential had stabilized within ± 1 mV prior to data acquisition.

#### Selectivity of the fabricated sensor for copper ions

The selectivity of the Cu²⁺-selective sensor was evaluated by measuring its potential response in the presence of potential interfering ions, including Hg²⁺, Ca²⁺, K⁺, Mg²⁺, and Na⁺. Selectivity coefficients, $\:\mathrm{log}\left({\mathrm{k}}_{{\mathrm{C}\mathrm{u}}^{2+},\:\mathrm{I}\mathrm{n}\mathrm{t}\mathrm{e}\mathrm{r}\mathrm{f}\mathrm{e}\mathrm{r}\mathrm{e}\mathrm{n}\mathrm{t}\:\mathrm{i}\mathrm{o}\mathrm{n}\mathrm{s}}^{\mathrm{p}\mathrm{o}\mathrm{t}}\right)$, were calculated using the Separate Solution Method (SSM) according to IUPAC guidelines [36], to quantify the sensor’s specificity toward Cu²⁺ ions in the presence of competing species. The SSM was performed based on ionic activities to account for the effective concentration of ions in solution, influenced by factors such as ionic strength and ion interactions.

The experimental procedure involved preparing two separate solutions: one containing 1 × 10⁻³ mol L⁻¹ Cu²⁺ ions of high purity with no interfering ions, and the other containing 1 × 10⁻³ mol L⁻¹ of each interfering ion (e.g., Hg²⁺, Ca²⁺, etc.) with no Cu²⁺. The Cu²⁺-selective electrode and a double-junction Hg/HgCl₂ calomel reference electrode were immersed in each solution, and the potentials (E₁ for Cu²⁺ and E₂ for each interfering ion) were recorded after stabilization within ± 1 mV using a JENWAY 3505 pH/mV/Temperature Meter. The selectivity coefficient was determined using the following equation:

$$\begin{gathered}\mathrm{log}\left({\mathrm{k}}_{{\mathrm{C}\mathrm{u}}^{2+},\:\mathrm{I}\mathrm{n}\mathrm{t}\mathrm{e}\mathrm{r}\mathrm{f}\mathrm{e}\mathrm{r}\mathrm{e}\mathrm{n}\mathrm{t}\:\mathrm{i}\mathrm{o}\mathrm{n}\mathrm{s}}^{\mathrm{p}\mathrm{o}\mathrm{t}}\right) =\:\frac{{\mathrm{E}}_{2}-{\mathrm{E}}_{1}}{\mathrm{S}} \hfill \\ \hfill \qquad \qquad \qquad \qquad \hfill \qquad +\:\left(1-\frac{\mathrm{A}}{\mathrm{B}}\right)\:\mathrm{l}\mathrm{o}\mathrm{g}\:\mathrm{a}\mathrm{A}\end{gathered}$$

In this context, log $\left({\mathrm{k}}_{{\mathrm{C}\mathrm{u}}^{2+},\:\mathrm{i}\mathrm{n}\mathrm{t}\mathrm{e}\mathrm{r}\mathrm{f}\mathrm{e}\mathrm{r}\mathrm{e}\mathrm{n}\mathrm{t}\:\mathrm{i}\mathrm{o}\mathrm{n}\mathrm{s}}^{\mathrm{p}\mathrm{o}\mathrm{t}}\right)$ represents the selectivity coefficient, where S denotes the slope of the Cu^2⁺^ SC-ISE, determined from the calibration curve. E_1_​ refers to the potential recorded in 1 × 10⁻³ mol L⁻¹ solution of Cu^2⁺^ ions, while E_2_​ represents the potential measured in 1 × 10⁻³ mol L⁻¹ solution of interfering ions. A and B correspond to the charges of copper ions and interfering ions, respectively, and a indicates the activity of Cu^2⁺^ ions. Measurements were conducted in triplicate.

#### Preparation of copper nanoparticles

A quantity corresponding to 0.125 g of CuCl₂·2 H₂O was dissolved in 50 mL of deionized water to prepare a 10⁻² mol L⁻¹ CuCl₂·2 H₂O solution. The resulting solution was heated to 80 °C in an oil bath under continuous magnetic stirring. Subsequently, 50 mL of an aqueous L-ascorbic acid solution (0.2 mol L⁻¹) was added dropwise to the reaction vessel while maintaining constant agitation. The mixture was maintained at 80 °C until a dark-colored solution was observed, indicating the progression of the reaction [37]. 

#### Characterization of nanoparticles

The UV-Vis absorption spectra of the synthesized dispersions were recorded using a Shimadzu dual-beam UV-Vis spectrophotometer. The morphology and particle size of the prepared Cu²⁺ nanoparticles were characterized by transmission electron microscopy (TEM). Furthermore, Fourier-transform infrared (FTIR) spectroscopy was employed to analyze the functional groups present in the samples.

### Inline tracking of Cu^+2^ decrease during the synthesis of copper nanoparticles: a kinetic study

Potentiometric measurements were performed using a JENWAY digital analyzer configured for continuous monitoring of potential variations. A CuCl₂·2 H₂O solution was prepared by dissolving 10 mmol of CuCl₂·2 H₂O in 50 mL of deionized water. The solution was heated to 80 °C in an oil bath under constant magnetic stirring, and a thermometer was placed in the reaction flask to ensure accurate temperature monitoring throughout the experiment. Both the Hg/HgCl₂ calomel reference electrode and the Cu²⁺-selective working electrode were immersed in the reaction medium. Subsequently, 50 mL of L-ascorbic acid solution prepared at varying concentrations (0.4, 0.6, 0.8, and 1.0 mol L⁻¹) was added dropwise to the flask under continuous stirring. The change in potential was recorded throughout the reduction process until stabilization within ± 1 mV, with the appearance of a dark solution confirming the formation of copper nanoparticles.

The experiment was repeated using identical reagent concentrations at reduced temperatures of 60 °C and 70 °C. The rate constant (k) for copper ion reduction and the activation energy (Ea) associated with nanoparticle formation were calculated using an Arrhenius plot. In addition, thermodynamic parameters—including the Gibbs free energy of activation (ΔG), entropy change (ΔS), and enthalpy change (ΔH) were derived based on the Eyring equation.

### Real-time monitoring of Cu²⁺ ion release during the decomposition of copper nanoparticles

The same experimental setup described earlier was employed to monitor the dissolution of synthesized copper nanoparticles. Two experiments were conducted to evaluate the stability and dissolution behavior of Cu nanoparticles.

In the first experiment, the reference and working electrodes were immersed in the prepared Cu nanoparticle dispersion, which was stored at −2 °C in a refrigerator for three days. During this period, the change in potential was continuously recorded to assess the stability of the Cu nanoparticles under these conditions.

In the second experiment, the oxidative degradation of copper nanoparticles and the consequent release of Cu²⁺ ions were induced using 0.05 mol L⁻¹ hydrogen peroxide (H₂O₂). Approximately 1 µg mL⁻¹ of the purified Cu nanoparticles was transferred to a beaker, into which both the reference and working electrodes were immersed. H₂O₂ was added dropwise to the nanoparticle dispersion while continuously monitoring the potential. This setup enabled real-time observation of the oxidation process and facilitated analysis of the Cu²⁺ ion release kinetics.

### Real water sample analysis

Four types of water samples were investigated: industrial wastewater, Nile River water, swimming pool water, and tap water. Nile River samples were collected from Giza, Egypt, following standard sampling procedures. Tap, pool, and river samples were collected from different sites in Cairo, while industrial wastewater was kindly provided by a local pharmaceutical plant in Cairo, Egypt. All samples were collected in pre-cleaned plastic bottles, labeled with sampling details (name, date, location), and transported to the laboratory in cooled containers to minimize compositional changes [38, 39].

Prior to analysis, samples were filtered through 0.45 μm membrane filters (Cosmonice Filter S, Nacalaitesque) to remove suspended particulates. Each sample was first analyzed directly (unspiked) to assess the natural baseline copper ion content. Subsequently, samples were spiked with CuCl₂ to a final concentration of 1 × 10⁻⁴ mol L⁻¹ to evaluate the recovery performance of the developed method.

For all measurements, the fabricated Cu²⁺-selective electrode and the reference electrode were immersed in the prepared water samples, and the electromotive force (EMF) was recorded. The unspiked analysis allowed determination of the inherent Cu²⁺ levels (or confirmation of concentrations below the detection limit), while the spiked analysis enabled calculation of recovery values to validate the method’s accuracy and applicability in real matrices.

## Results and discussion

Motivated by the need to develop a Cu²⁺-selective sensor for monitoring the formation, stability, and degradation of copper nanoparticles, as well as for tracking copper contamination in environmental samples, this work presents a successful approach for the fabrication, optimization, and application of a novel Cu²⁺ selective SC-ISE. The proposed sensor is simple, portable, rapid, and highly selective, enabling real-time tracking of Cu²⁺ concentration changes during the synthesis, storage, and decay of copper nanoparticles. Furthermore, the sensor was successfully applied to detect copper contamination in various water samples, including pool water, tap water, river water, and industrial wastewater.

### Fabrication of the Cu²^+^-selective membrane

The fabricated Cu²⁺-selective SC-ISE membrane exhibited excellent perm-selectivity and ion-exchange capabilities, specifically toward copper ions (Cu²⁺). Extensive research has demonstrated that the selectivity and sensitivity of ion-selective membranes are largely dictated by their compositional elements, including the plasticizer, ionophore, polyvinyl chloride (PVC) matrix, and ion exchanger [40, 41]. Accordingly, the membrane cocktail components were carefully selected to ensure high specificity for Cu²⁺ ions, enabling the sensor to achieve rapid response, enhanced sensitivity, and superior selectivity.

Polyvinyl chloride (PVC) was employed as the principal matrix material due to its high lipophilicity (Log *P* = 0.593) and its proven capacity to uniformly incorporate all membrane constituents. Moreover, PVC contributes to an elevated dielectric constant within the membrane, which facilitates increased ionic conductivity and supports the accumulation of higher ionic concentrations [42]. 

To determine the most effective plasticizer for membrane liquefaction, three candidates were evaluated—dioctyl phthalate (DOP), 2-nitrophenyl octyl ether (2-NPOE), and dibutyl sebacate (DBS)—representing different dielectric constant ranges (ε ≈ 5.1, 24, and 4.4, respectively). The dielectric constant of the plasticizer critically affects ion mobility and membrane permittivity, thereby influencing electrode sensitivity and response stability. Among the tested plasticizers, DOP exhibited the highest sensitivity and most stable Nernstian slope for Cu²⁺ detection. Its moderate dielectric constant provided an optimal balance between ionic transport and membrane homogeneity, minimizing baseline noise observed with 2-NPOE (high ε) and the reduced ionic mobility seen with DBS (low ε). Consequently, DOP was selected as the optimal plasticizer for achieving reproducible and efficient membrane performance [43].

Calix[6]arene and β-cyclodextrin (β-CD), act as molecular receptors ionophores for specific analytes, including copper ions (Cu²⁺). Their ability to selectively recognize and bind target molecules is based on the formation of inclusion complexes and formation of hydrogen or ionic bonds [43]. Calix[6]arene and β-CD were evaluated for incorporation into the membrane cocktail as ionophores for Cu²⁺. β-CD demonstrated superior performance compared to Calix[6]arene, likely due to its unique structural characteristics. β-CD comprises seven glucose monomers arranged in a cone-shaped ring, forming a hydrophobic cavity that is well-suited for the inclusion of divalent cations like Cu²⁺. Copper ions, with a hydrated radius of approximately 4.2 Å, can be effectively accommodated within β-CD’s internal cavity, which has a diameter of about 7–8 Å. β-CD functions as an artificial receptor for Cu²⁺ ions by binding them to the electron-rich oxygen atoms of its glucopyranose rings within the hydrophobic cavity [44]. This binding mechanism enables the selective encapsulation of Cu²⁺ ions. Furthermore, the immobilized cavity of β-CD retains its complexation ability, ensuring stable inclusion complexes with other molecules while maintaining its structural integrity [45]. These properties make β-CD a good choice for enhancing the selectivity and sensitivity of the Cu²⁺ ion-selective membrane. Ion exchangers are incorporated into the membrane matrix to provide mobile ionic sites, facilitating interfacial ion exchange and reducing ionic resistance [46]. 

Copper ions (Cu²⁺), as divalent cations, necessitate a membrane exhibiting cation-exchange functionality to facilitate selective interaction. This requirement was addressed by incorporating a lipophilic cationic exchanger specifically Reinecke’s salt into the membrane formulation [47]. Prior to use, the fabricated Cu²⁺-selective sensor was conditioned in a 1 × 10⁻² mol L⁻¹ CuCl₂·2 H₂O solution for several hours to promote the exchange of the ammonium ions (NH₄⁺) present in Reinecke’s salt with Cu²⁺ ions.

To identify the most suitable cationic exchanger, various alternatives were tested, including Reinecke’s salt, tetraphenylborate (TPB), and phosphotungstic acid (PT). Preliminary assessments revealed that membranes prepared with TPB and PT exhibited slope values of 31.62 and 29.07 mV/decade, respectively, with correlation coefficients of 0.9987 and 0.9990. Despite these values, sensors incorporating TPB and PT displayed lower sensitivity, non-reproducible responses, and reduced operational stability. Conversely, membranes containing Reinecke’s salt produced a consistent Nernstian slope, rapid and stable responses, and superior detection limits.

The calculated molar ratio of the ionophore (β-cyclodextrin, β-CD) to the ion exchanger (Reinecke’s salt) was approximately 1.7:1, indicating that the ion exchanger constituted nearly 50 mol% relative to the ionophore. This proportion is consistent with literature reports, which suggest that for divalent cations, an optimal performance is achieved when the concentration of anionic sites is around 50 mol% of the ionophore content [48]. These results further support the effective formation of an inclusion complex between β-CD and Cu²⁺ ions, contributing to the enhanced performance of the developed sensor.

### Polyaniline as an ion-to-electron transducer layer in SC-ISE

Among the class of electrically conducting polymers (CPs), polyaniline (PANI) is particularly distinguished by its diverse synthesis routes—both electrochemical and chemical as well as the exceptional stability of its electrically conductive emeraldine salt (ES) form. Nanostructured variants of PANI, including nanoparticles, nanorods, and nanowires, have attracted considerable interest in recent research due to their distinctive physicochemical characteristics that contribute to enhanced functional performance. One of the key advantages PANI holds over other CPs lies in its processability, as it can be formulated into soluble forms and stable dispersions, thereby broadening its scope of application [49]. 

From a potentiometric standpoint, the environmental robustness and ease of processing of PANI nanoparticles render them highly suitable as an Ion-to-electron transducer layer in SC-ISE. The enhanced potentiometric stability provided by nanostructured PANI is primarily due to the extensive interfacial contact between the ion-selective membrane and the electrically conductive nanomaterial, which promotes the formation of a large electrical double-layer capacitance. This capacitive interface contributes significantly to stabilizing the electrode potential. In this context, PANI nanoparticles function as a hydrophobic intermediate transducer layer, positioned between a β-CD-doped PVC membrane and a glassy carbon electrode, thereby enabling stable and reliable potential measurements.

Previous designs of SC-ISE that incorporated electropolymerized PANI layers typically employed aqueous solutions of either HCl or H₂SO₄. However, these methods introduced water into the solid-contact layer from the initial stages of the electrode’s operation. In certain instances, washing the electropolymerized PANI layer with distilled water led to partial conversion of the conducting emeraldine salt (ES) form to the non-conducting emeraldine base (EB) form. This transition can compromise the reproducibility of the standard potential due to the heightened sensitivity of electrodeposited PANI to H⁺ ion activity [50]. A potential approach to mitigate these issues and eliminate the introduction of water during electrode preparation is to utilize dispersions of conducting polymers (CPs) that dissolve in organic solvents.

In this study, polyaniline nanoparticles stabilized with sodium dodecyl sulfate (PANI SDS) were synthesized via a chemical polymerization method adapted from Han et al. [51] The dispersion polymerization of aniline was carried out at 20 °C, with SDS acting as both the dopant and stabilizer. Ammonium persulfate (APS) was employed as the initiator oxidant. After 2.5 h of polymerization, the reaction produced dark green emeraldine PANI SDS, confirming the formation of the conducting emeraldine salt form of PANI. To achieve a high degree of doping, a 1:1 monomer-to-dopant (SDS) ratio was used. The PANI nanoparticles were extensively characterized using various spectroscopic techniques, and their particle size was measured, ensuring their suitability for use as a transducer layer in SC-ISE.

### Characterization of polyaniline nanoparticles using UV/VIS

Spectroscopic techniques have been widely employed to characterize conducting polymers and to identify their conductive species, such as polarons and bipolarons. To analyze the chemical composition of PANI–SDS, UV–Vis absorption spectroscopy was conducted. The absorption spectrum of the PANI–SDS dispersion (at pH < 2) confirmed the presence of the conducting emeraldine salt (ES) form. The spectrum displayed characteristic polaron bands at 785 nm and approximately 420 nm, in addition to a π–π* transition band around 350 nm. The overlap of the π–π* transition band at 350 nm further supports the identification of the conducting form of the polymer.

### Effect of change of pH on the copper selective sensor performance

**Fig. 1:**
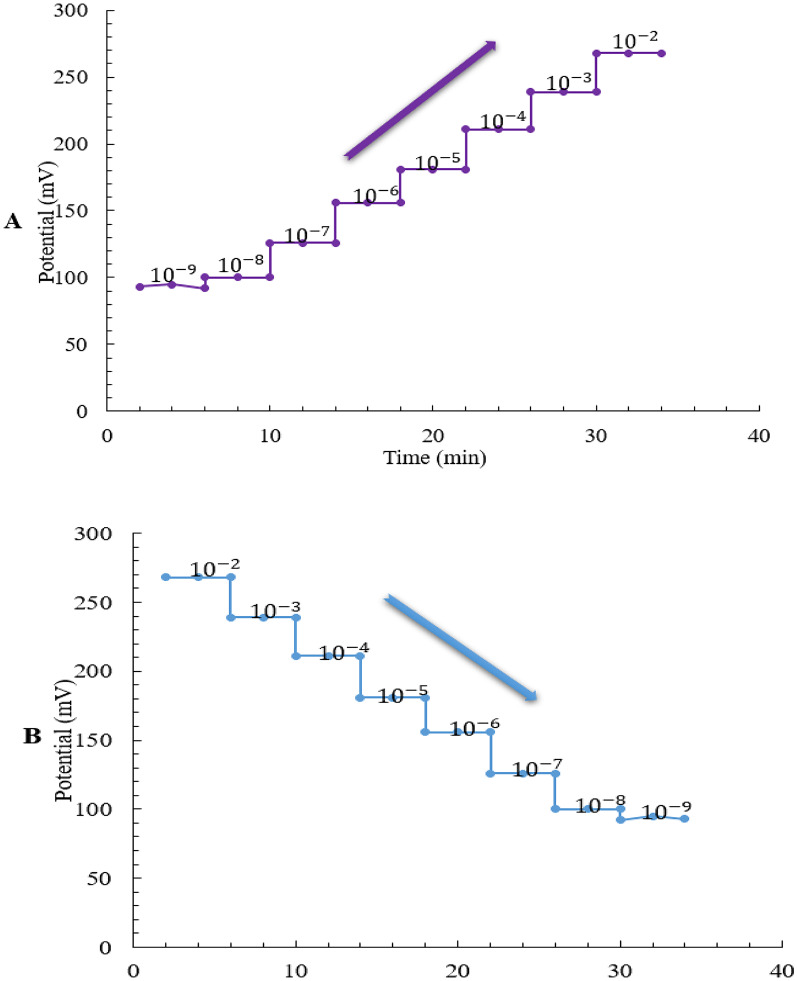
Change of potential with time during (**a**) moving from lower to higher copper ion concentration and (**b**) moving from higher to lower copper ion concentration

The effect of pH on the electrode performance was evaluated, and the results are shown in Figure (1s). The membrane potential remained stable within the pH range of 6–9, confirming reliable sensor performance under these conditions. At pH values below 2, a decrease in potential was observed due to competition from excess H⁺ ions. Beginning around pH 6, copper undergoes stepwise hydrolysis, forming soluble species such as CuOH⁺ and Cu₂(OH)₂²⁺. Although these complexes slightly reduce the activity of free Cu²⁺, the electrode response remained stable within the working range. At pH values above 9, however, extensive hydrolysis leads to the formation of insoluble Cu(OH)₂, which significantly decreases the free Cu²⁺ concentration in solution and causes potential drift.

The wide stability range (pH 6−9) is advantageous for the accurate and reproducible quantification of copper ions. Additionally, the synthesis and degradation experiments of citrate-stabilized copper nanoparticles (Cit-CuNPs) were performed in bi-distilled water, which falls within this pH range, ensuring the sensor’s applicability for real-time monitoring.

### Dynamic response characteristics of the electrode

The dynamic response characteristics of the Cu²⁺-selective electrode were evaluated by monitoring the potential variation of the fabricated Cu²⁺ selective SC-ISE upon sequential changes in copper ion concentrations, both in ascending and descending order, as illustrated in Fig. 1. The electrode exhibited a response time of approximately 10–12 s to reach a stable potential within ± 1 mV, indicating its effectiveness in real-time monitoring of fluctuations in Cu²⁺ ion activity.

### The method calibration, accuracy, precision, and repeatability

**Fig. 2:**
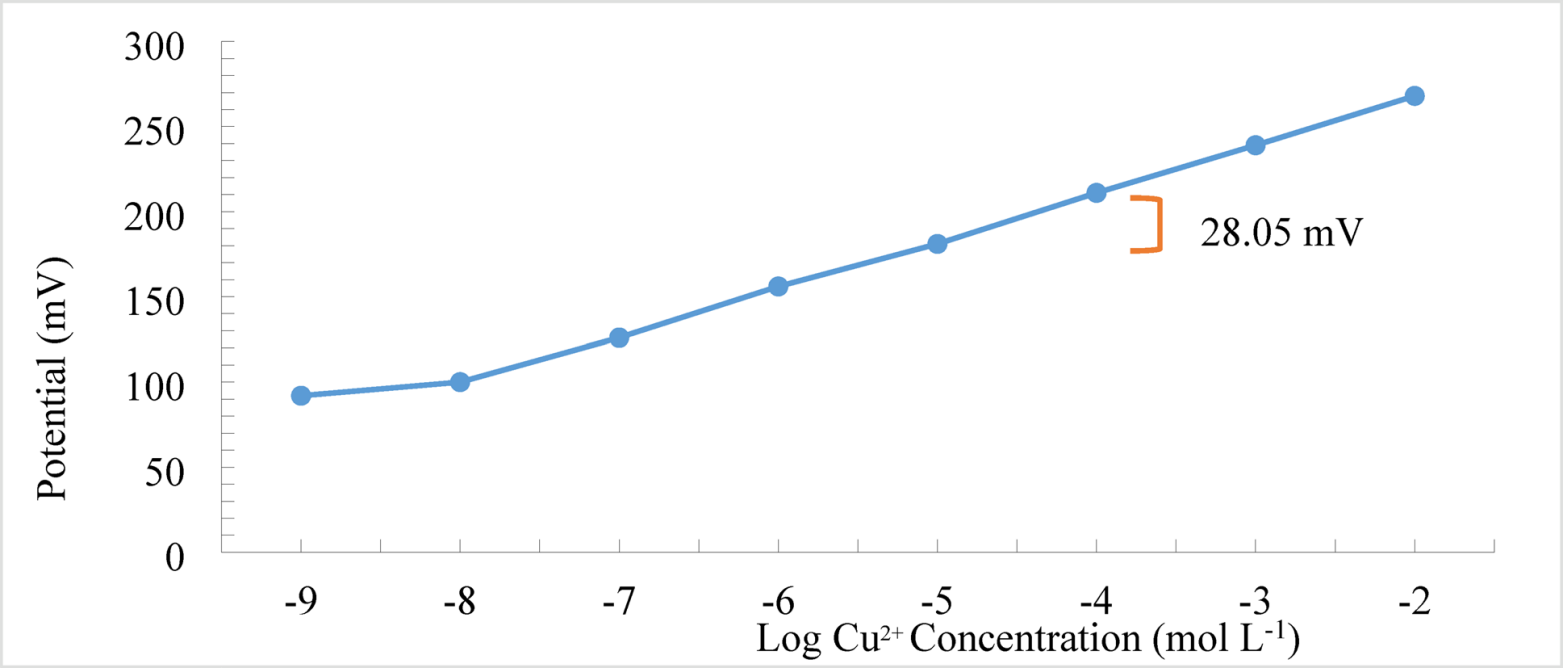
Calibration curve of potential (mV) versus log copper ion concentration

The calibration curve shown in Figure 2 reveals a linear potentiometric response over the Cu²⁺ concentration range of 1 × 10⁻⁸ to 1 × 10⁻² mol L⁻¹, exhibiting a consistent Nernstian slope of 28.05 mV per decade of activity. The sensor demonstrated a detection limit of 7.5 × 10⁻⁹ mol L⁻¹. Method accuracy was verified through the analysis of blind standard solutions at concentrations of 1 × 10⁻⁵, 1 × 10⁻⁴, and 1 × 10⁻³ mol L⁻¹ CuSO₄ under optimized experimental conditions. Precision was evaluated by assessing both repeatability (intra-day) and intermediate precision (inter-day). The Cu²⁺ concentrations were calculated using the derived regression equation. As presented in Table 1, the results confirmed the method’s high accuracy and precision, with relative standard deviation (RSD) values consistently below 2%. Furthermore, the mean recovery values ranged from 98% to 102%, highlighting the method’s robustness and suitability for routine analytical applications.

Reproducibility was assessed by examining the effect of several factors such as changing the potentiometer used for analysis, usage of Ag/AgCl2 reference electrode instead of Calomel reference electrode, or fabricating multiple electrodes using independently prepared membrane batche using the same membrane components from different companies. The consistent slope (28.05 mV/decade) and RSD < 2% in accuracy and precision data confirm high reproducibility among the prepared electrodes.

**Table 1:** Metrological and assay validation parameters for copper ions using the fabricated copper selective electrode

Parameter	Cu^+2^ SC-ISE
*Metrological parameters*	
Concentration range (mol L⁻¹)	1 × 10^− 8^ – 1 × 10^− 2^
Slope (mV/decade)^a^	28.05
Intercept (mV)	323.15
Correlation coefficient	0.9996
Working pH	6–9
Response time (S)	≤ 10
Stability	At least two months
*Assay validation parameters*	
Accuracy (Mean ± S.D)^b^	99.28 ± 1.22
*Precision (RSD %)*	
Repeatability^c^	0.866
Intermediate precision^d^	0.928
LOQ (mol L⁻¹)	2.6 × 10^− 8^
LOD (mol L⁻¹)^e^	7.5 × 10^− 9^

^a^Average of three determination

^b^Average of three determination using the concentration of (1 × 10^− 5^, 1 × 10^− 4^ and 1 × 10^− 3^ mol L⁻¹)

^c^The RSD% obtained after the intraday repeating the same concentrations

^d^The RSD% obtained after the interday repeating the same concentrations

^e^Limit of detection calculated by the interception of the extrapolated arms of the responsive Nernstian segment of the calibration graph and the nonresponsive arm

### **The fabricated electrode selectivity towards copper ions**

The selective permeability of ion-selective membranes toward copper ions is quantitatively expressed by the potentiometric selectivity coefficient, log $\left({\mathrm{k}}_{{\mathrm{C}\mathrm{u}}^{2+},\:\mathrm{i}\mathrm{n}\mathrm{t}\mathrm{e}\mathrm{r}\mathrm{f}\mathrm{e}\mathrm{r}\mathrm{e}\mathrm{n}\mathrm{t}\:\mathrm{i}\mathrm{o}\mathrm{n}\mathrm{s}}^{\mathrm{p}\mathrm{o}\mathrm{t}}\right)$. This parameter is affected by various experimental conditions, including temperature, the ionic strength of the solution, and the concentrations of interfering ions. In the present study, the membrane composition was strategically optimized to enhance Cu²⁺ selectivity, primarily through the incorporation of β-cyclodextrin (β-CD), a ligand known for its high affinity toward copper ions [52]. The selectivity performance of the developed sensor was evaluated via the separate solutions method, which measures the electrode potential in individual solutions of the primary and interfering ions. As in Table 2, the sensor exhibited a good response towards Cu²⁺ while showing minimal interference from other tested metal ions. These findings confirm the sensor’s excellent selectivity and its potential for accurate determination of copper ions in complex matrices.

**Table 2:** Logarithmic selectivity coefficient, log $\left({\mathrm{k}}_{{\mathrm{C}\mathrm{u}}^{2+},\:\mathrm{i}\mathrm{n}\mathrm{t}\mathrm{e}\mathrm{r}\mathrm{f}\mathrm{e}\mathrm{r}\mathrm{e}\mathrm{n}\mathrm{t}}^{\mathrm{p}\mathrm{o}\mathrm{t}}\right)$, using the separate solutions method

Interferent ion	log $\left({\mathbf{k}}_{{\mathrm{C}\mathrm{u}}^{2+},\:\mathbf{i}\mathbf{n}\mathbf{t}\mathbf{e}\mathbf{r}\mathbf{f}\mathbf{e}\mathbf{r}\mathbf{e}\mathbf{n}\mathbf{t}}^{\mathbf{p}\mathbf{o}\mathbf{t}}\right)$^a^
Zinc (Zn²⁺)	−5.67
Nickel (Ni²⁺)	−6.21
Cobalt (Co²⁺)	−6.89
Iron (Fe²⁺)	−6.45
Manganese (Mn²⁺)	−7.02
Lead (Pb²⁺)	−6.78
Magnesium (Mg²⁺)	−7.34
Sodium (Na⁺)	−7.89

^a^Average of three measurements

### The lifetime and the reuse of the proposed electrode

The operational stability of the fabricated electrode was assessed over a two-week period by continuously measuring Cu²⁺ concentrations and extended up to two months of intermittent measurements. During the first month time, the electrode exhibited stable performance, with no significant deviation in the response slope, and a relative standard deviation (RSD) of only 1.8%. However, after one month of use, a gradual decline in performance was observed, with the response slope decreasing to 75% of its initial value at the end of two months, indicating a gradual loss of sensitivity.

To restore the electrode’s original performance, two regeneration approaches were investigated: Surface Polishing: The electrode surface was mechanically polished to remove the upper degraded layer, exposing a fresh, functional surface. Following this treatment, the response slope recovered to 95.1% of its initial value, demonstrating that mechanical renewal can effectively restore electrode activity. Electrochemical Regeneration: The used electrode was subjected to a constant potential reduction in 0.2 mol L^− 1^ NaCl solution at − 0.5 V for 30 min. This electrochemical treatment successfully regenerated the electrode, restoring the response slope to 97.9% of its initial value.

### Potentiometric aqueous layer test

Although the PANI-based SC-ISE exhibited strong short-term stability and reliable potentiometric responses for Cu²⁺ detection, concerns regarding their long-term stability remain—particularly the potential formation of an aqueous layer at the interface between the solid contact and the ion-selective membrane. To investigate this issue, a potentiometric aqueous layer test was performed in accordance with established methodologies [53] This test evaluates potential drifts caused by the accumulation or displacement of ions within a possible interfacial aqueous layer. Specifically, the electrode was first immersed in a 0.1 mmol L⁻¹ Cu²⁺ solution, followed by exposure to a 10 mmol L⁻¹ solution of an interfering ion, and subsequently returned to the original copper solution. The presence of a water layer is indicated by irreversible potential changes arising from shifts in the composition of the interfacial solution due to ion exchange and diffusion processes. In this context, Ni²⁺ was chosen as the interfering species owing to its divalent nature and coordination characteristics, which closely resemble those of Cu²⁺ and may influence the electrode’s response.

As shown in Figure (2s), no significant potential drift was observed for the Cu²⁺-selective electrode, indicating the absence of an aqueous layer beneath the membrane. This finding is consistent with previous reports demonstrating that the incorporation of highly hydrophobic PANI nanoparticles effectively prevents water layer formation, thereby improving the long-term stability of the Cu²⁺-selective electrode.

### Copper nanoparticles characterization

**Fig. 3:**
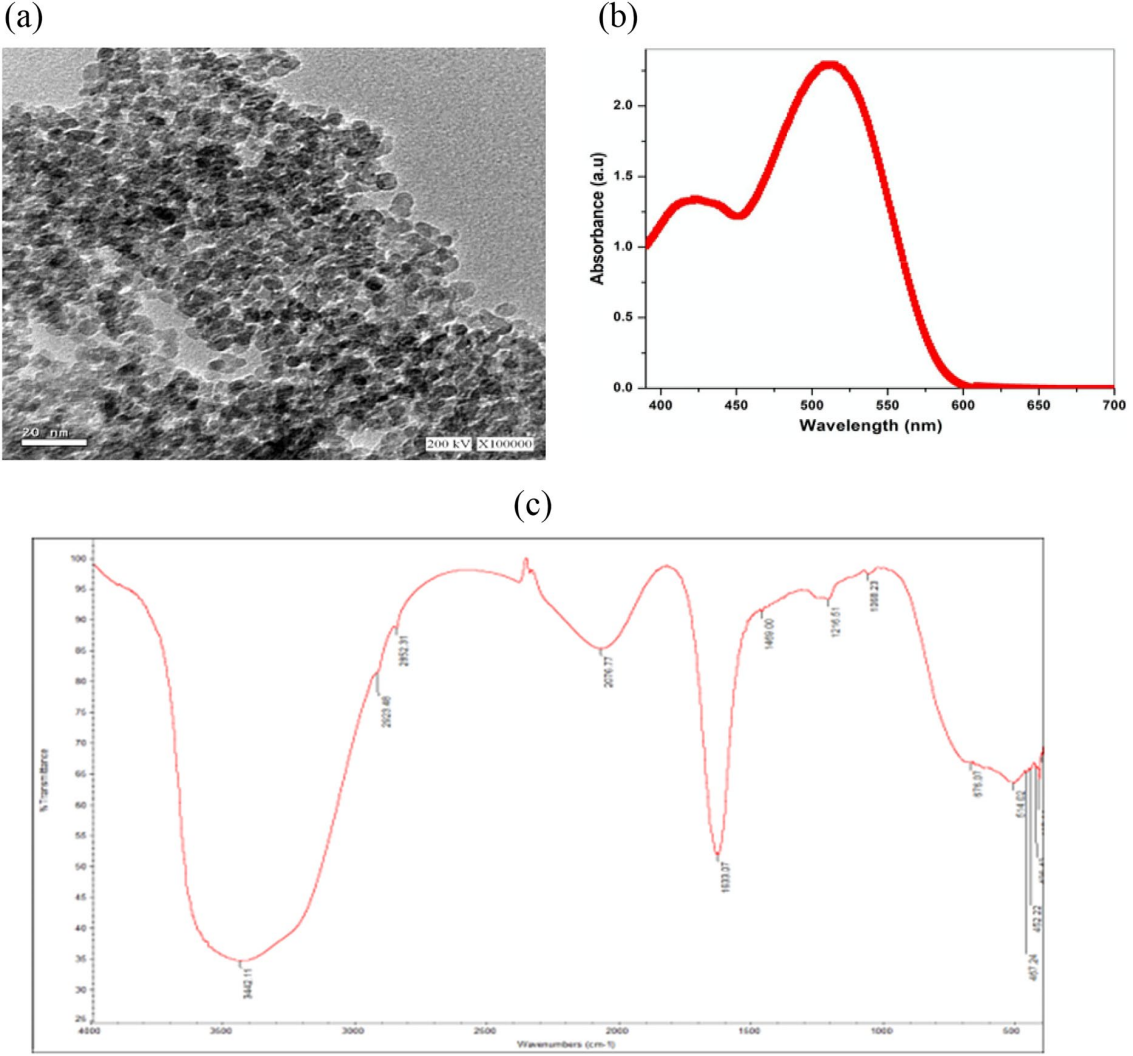
Copper nanoparticles characterization by **a**: transmission electron microscope, **b**: UV spectrophotometer, **c**: FTIR spectrometer

The size of copper nanoparticle and its shape were confirmed using the transmission electron microscope which showed spherical crystalline particles as described in the literature. UV spectrum was recorded from 200 to 800 nm with a maximum peak showing at approximately 525 nm, while the FTIR revealed two peaks at 3442 cm^− 1^ and 1633 cm^− 1^ as in Figure 3. All results confirmed the formation of separate copper particles in the nano range [54, 55].

### Real-time inline tracking of copper nanoparticles synthesis

Copper nanoparticles (CuNPs) were synthesized using the method of chemical reduction using CuCl₂·2 H₂O as the precursor and L-ascorbic acid as the reducing agent [37, 56]. The reduction process followed a stepwise mechanism, where Cu²⁺ ions were initially reduced to Cu⁺, which subsequently underwent further reduction to form zero-valent copper (Cu⁰). This transformation was accompanied by nucleation and growth processes, ultimately leading to the formation of colloidal CuNPs. While L-ascorbic acid primarily acted as the reductant, it also transiently interacted with the nanoparticle surface, providing stabilization. The overall reaction mechanism can be expressed as:

$${\mathrm{C}}{{\mathrm{u}}^{{\mathrm{2}}+}}+{\text{ 2}}{{\mathrm{C}}_{\mathrm{6}}}{{\mathrm{H}}_{\mathrm{8}}}{{\mathrm{O}}_{\mathrm{6}}} \to {\text{ C}}{{\mathrm{u}}^0}\,+\,{\mathrm{2}}{{\mathrm{C}}_{\mathrm{6}}}{{\mathrm{H}}_{\mathrm{6}}}{{\mathrm{O}}_{\mathrm{6}}}\,+\,{\mathrm{2}}{{\mathrm{H}}^+}$$

To achieve real-time monitoring of CuNP formation, a copper ion-selective electrode (Cu-ISE) was employed, providing a selective and rapid response to the decrease of Cu²⁺ ions concentration during the reduction process. The electrode’s high sensitivity facilitated the tracking of Cu²⁺ concentration fluctuations throughout nanoparticle synthesis under varying thermal conditions. The reaction kinetics were analyzed by recording the potential variation over time at three different temperatures (60, 70, and 80 °C) while maintaining the precursor concentrations constant. This approach enabled a systematic evaluation of the temperature-dependent effects on copper nanoparticle formation and reaction dynamics.

**Fig. 4:**
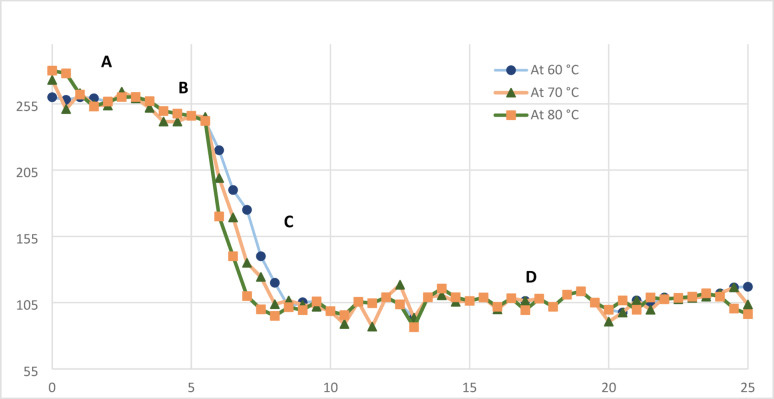
CuNPs profile synthesis showing the decrease of potential due to the reduction of Cu^+2^ concentration over time at different temperatures: **A** stable potential due to copper ions, **B** the point at which ascorbic acid injection began, **C** the decrease in potential at different temperatures due to reduction of Cu^+2^ concentration leading to the formation of copper nanoparticles, **D** the Nanoparticles growth and nucleation phase

Prior to each measurement, the potentiometric response of the electrode was optimized, and a fresh calibration was conducted at each temperature to ensure reliable and accurate readings. As illustrated in Figure 4, the kinetic profiles obtained at 60, 70, and 80 °C consistently displayed a sigmoidal trend, indicative of an autocatalytic mechanism governing the formation of copper nanoparticles. The shape of the curve reflects a characteristic four-step process typically associated with the nucleation and growth of metal nanoparticles, encompassing initial ion reduction, nucleation, surface catalyzed growth, and subsequent aggregation or stabilization.

In the initial phase (a), the potential remained stable upon immersing the Cu²⁺ ion-selective electrode in the CuCl₂ solution, indicating equilibrium conditions. Phase (b) corresponds to the initiation of the reduction reaction upon the dropwise addition of L-ascorbic acid, leading to a gradual decrease of Cu²⁺ ions. Subsequently, phase (c) is marked by a rapid potential drop, which is more observed at higher temperatures (80 °C) and less significant at lower temperatures (60 °C), reflecting an accelerated reduction rate due to enhanced reaction kinetics at elevated temperatures. Finally, in phase (d), the rate of potential change slows down, approaching a plateau, indicating the completion of Cu²⁺ ion reduction and the stabilization of the formed nanoparticles. Several studies on the formation of metallic nanoparticles, particularly silver and copper, in the presence of weak stabilizing agents have reported similar kinetic trends. The fluctuations in potential may be attributed to reorganization events, such as particle coalescence, Ostwald ripening, or secondary nucleation, which alter the ionic environment and subsequently affect the electrode response.

Ascorbate not only serves as a reducing agent in the synthesis of copper nanoparticles (CuNPs), but also temporarily adsorbs onto the nanoparticle surfaces. This adsorption can influence nucleation and growth pathways by modulating surface interactions and preventing uncontrolled aggregation. However, its stabilizing effect is generally weaker compared to dedicated capping agents like citrate or polymeric stabilizers [57]. 

The activation energy (Eₐ) associated with the reduction of Cu²⁺ ions during the formation of copper nanoparticles (CuNPs) was calculated based on the Arrhenius equation, as described in reference [56] :

$$\:\mathrm{k}=\mathrm{A}{\mathrm{e}}^{-{\mathrm{E}}_{\mathrm{a}}/\mathrm{R}\mathrm{T}} \to \mathrm{ln}\mathrm{k}=\frac{-{\mathrm{E}}_{\mathrm{a}}}{\mathrm{R}\mathrm{T}}\:+\mathrm{ln}\mathrm{A}$$

In this context, T denotes the absolute temperature in Kelvin, k is the rate constant, R is the universal gas constant (8.314 J mol⁻¹ K⁻¹), Eₐ represents the activation energy, and A corresponds to the frequency (pre-exponential) factor. A linear relationship was observed upon plotting ln k versus 1/T, as illustrated in Figure (3s). The activation energy (Eₐ) was derived from the slope of this line and calculated to be 34.06 kJ mol⁻¹, reflecting the minimum energy barrier necessary for the reduction reaction to proceed. The intercept of the linear plot yielded the frequency factor (A), which was found to be 9.3 × 10⁵ s⁻¹. This parameter represents the number of effective molecular collisions per unit time, where the orientation and energy are sufficient to facilitate product formation.

Various thermodynamic parameters, including the Gibbs free energy of activation (ΔG), entropy of activation (ΔS), and enthalpy of activation (ΔH), were calculated using the Eyring equation, which is expressed as follows:

$$\:\mathrm{ln}\frac{\mathrm{K}}{\mathrm{T}}=\:\left(-\frac{{\Delta\:}\mathrm{H}}{\mathrm{R}}\right)\left(\frac{1}{\mathrm{T}}\right)+\mathrm{ln}\frac{{\mathrm{K}}_{\mathrm{B}}}{\mathrm{h}}+\frac{{\Delta\:}\mathrm{S}}{\mathrm{R}}$$

where k is the rate constant, T is the absolute temperature (K), R is the universal gas constant (8.314 J mol⁻¹ K⁻¹), k_B_ is the Boltzmann constant (1.381 × 10⁻²³ J/K), and h is Planck’s constant (6.626 × 10⁻³⁴ J·s).

In this equation, k denotes the rate constant, T represents the absolute temperature (in Kelvin), R is the universal gas constant (8.314 J mol⁻¹ K⁻¹), k_B_ is the Boltzmann constant (1.381 × 10⁻²³ J·K⁻¹), and h is Planck’s constant (6.626 × 10⁻³⁴ J·s). These parameters collectively allow for the estimation of the energy and entropy changes associated with the transition state, thereby providing insights into the molecular dynamics of the Cu²⁺ reduction process.

Upon plotting $\left(\mathrm{ln}\frac{\mathrm{K}}{\mathrm{T}}\right)$ against $\:\left(\frac{1}{\mathrm{T}}\right)$, a linear relationship was observed as in Figure (4s). The slope of this plot corresponds to $\:\left(-\frac{{\Delta\:}\mathrm{H}}{\mathrm{R}}\right)$ from which the activation enthalpy ($\:{\Delta\:}\mathrm{H}$) was computed to be 31.21 KJ Mol^− 1^. The positive value of ΔH suggests that the reduction of CuCl₂ by ascorbic acid is an endothermic process, which absorbs energy from the surrounding solution. The entropy change (∆S) was determined from the intercept of the Eyring plot, given by $\left(\mathrm{ln}\frac{{\mathrm{K}}_{\mathrm{B}}}{\mathrm{h}}+\frac{{\Delta\:}\mathrm{S}}{\mathrm{R}}\right)$ and it was found to be −140.29 J mol^− 1^ K^− 1^. The negative value of ΔS implies that the copper nanoparticles, which are the reduction product, possess lower entropy compared to the reactants, indicating that the reaction is not spontaneous on its own and proceeds relatively slowly. This behavior is consistent with the ability of the process to be monitored potentiometrically.

The following equation used to calculate the activation free energy (∆G):

$$\:{\Delta\:}\mathrm{G}=\:{\Delta\:}\mathrm{H}-\mathrm{T}{\Delta\:}\mathrm{S}$$

The Gibbs free energy of activation (ΔG) values, calculated as 77.95 kJ mol⁻¹, 79.35 kJ mol⁻¹, and 80.75 kJ mol⁻¹ at 333 K, 343 K, and 353 K, respectively, indicate that the reduction of Cu²⁺ ions by L-ascorbic acid to form copper nanoparticles is a non-spontaneous process (ΔG > 0). This non-spontaneity implies that the reaction is thermodynamically unfavorable without external energy input. The elevated temperatures (60–80 °C) used in the experiments provide the necessary thermal energy to overcome the activation energy barrier (Eₐ = 34.06 kJ mol⁻¹), thereby accelerating the reaction kinetics and enabling nanoparticle formation. While this thermal energy drives the reaction forward, it does not alter the non-spontaneous nature of the process, as evidenced by the consistently positive ΔG values. The temperature dependence of the reaction rate, observed in the kinetic profiles (Fig. 4), highlights the role of heat in facilitating this endothermic, non-spontaneous process.

Based on these findings, it can be concluded that the developed Cu²⁺ SC-ISE is a promising candidate for investigating the synthesis kinetics of copper nanoparticles (CuNPs).

Although thermodynamic and kinetic parameters such as ΔH, ΔG, and Eₐ are traditionally determined by spectroscopic or calorimetric techniques, the potentiometric approach adopted here offers complementary advantages. Because the electrode potential directly reflects the logarithmic activity of ionic species at the membrane interface, real-time potential, time data can be used to derive reaction rate constants and, subsequently, activation and thermodynamic parameters. This electrochemical pathway captures the energy profile of the reduction process under its actual reaction conditions without the need for sample withdrawal or external perturbation. While potentiometry does not measure absolute heat changes as calorimetry does, it provides in-situ dynamic information that links ionic activity, redox kinetics, and temperature dependence within a single experimental setup. The good linearity of both Arrhenius and Eyring plots (Figs. 3s and 4s) validates the reliability of this electrochemical estimation method.

### Real-time inline monitoring of copper ion release during copper nanoparticles decay

Copper nanoparticles exhibit stability in aqueous solutions under standard conditions, and their oxidative dissolution, leading to the release of copper ions (Cu²⁺), is typically induced by oxidizing agents like oxygen or hydrogen peroxide. The fabricated Cu²⁺-selective sensor was effectively utilized for real-time monitoring of Cit-CuNPs degradation, both during storage in the refrigerator to maintain stability and upon stimulated release using H₂O₂.

Prior to each measurement, the potentiometric performance of the electrode was optimized, and calibration was conducted. To evaluate the stability of copper nanoparticles during storage, the Cu²⁺-selective electrode SC-ISE and reference electrode were placed in a beaker containing freshly prepared nanoparticles. The beaker was stored in a refrigerator at −2 °C, and the electrodes were connected to a potentiometer for continuous monitoring of potential variations over the course of one week. The nanoparticles demonstrated excellent stability, with only a minor increase in potential, likely resulting from minimal Cu²⁺ ion release, thereby confirming the robust stability of CuNPs at low temperatures (−2 °C). These results were further corroborated by intermittent measurements of particle size and zeta potential, both showing high stability.

**Fig. 5:**
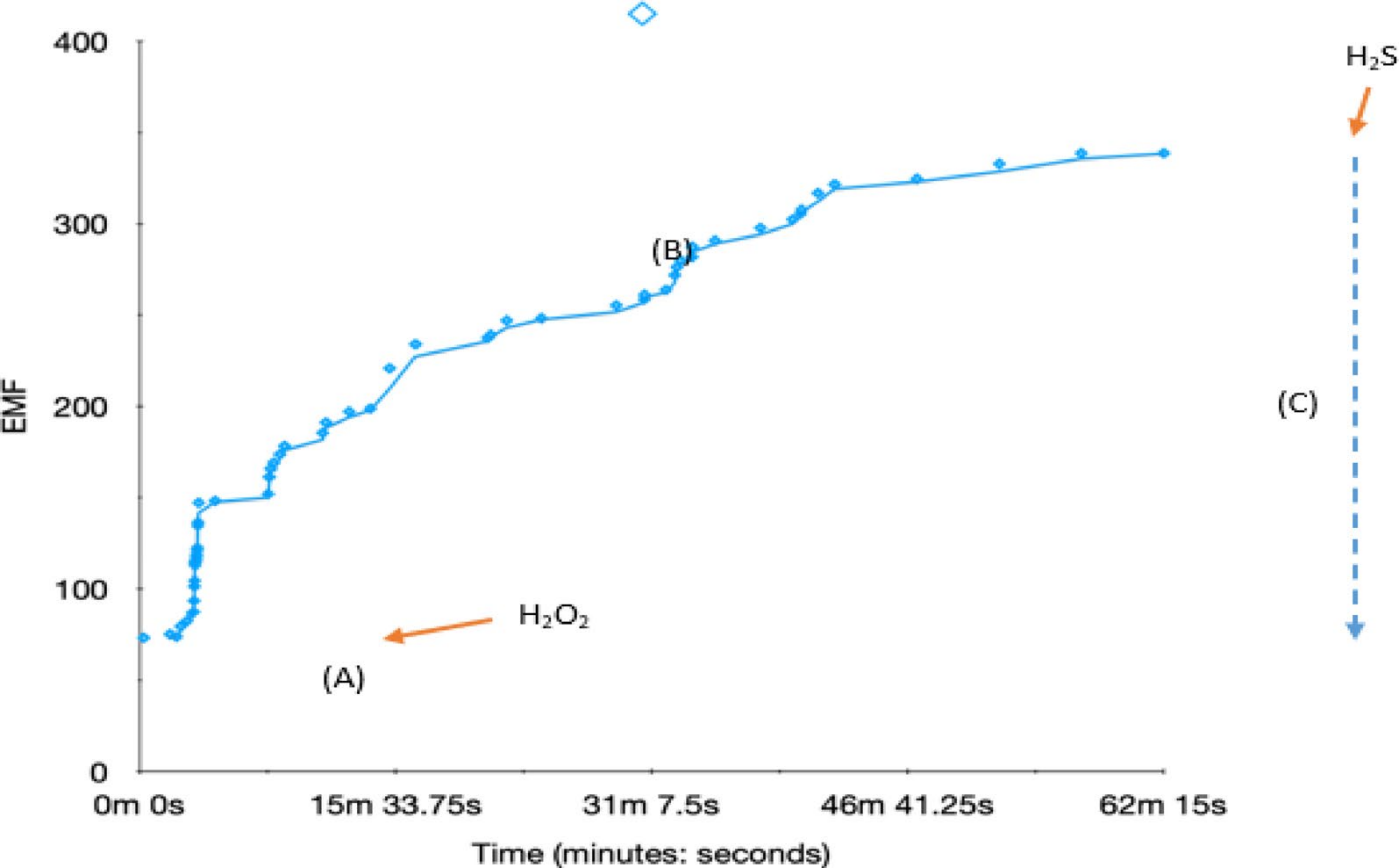
Inline monitoring of the change in copper ion concentration upon release from decayed Cit-AgNPs due to the addition of hydrogen peroxide

To induce the oxidative dissolution of copper nanoparticles and trigger the release of Cu²⁺ ions, hydrogen peroxide (H₂O₂) was utilized, as in Figure 5. In this experiment, the Cu²⁺-selective SC-ISE and calomel reference electrode were immersed in a beaker containing 25 mL of deionized water for continuous potential monitoring. After the potential stabilized, approximately 25 µg of CuNPs was introduced into the solution. Initially, a rapid increase in potential was observed due to the presence of residual copper ions from the CuNPs. Following this, the potential stabilized (a). When H₂O₂ was added, the potential rose further, indicating the oxidation of CuNPs and the subsequent release of free Cu²⁺ ions, as shown by arrow (b). Eventually, the potential reached a plateau. The addition of concentrated hydrogen sulfide (H₂S) led to the formation of a black precipitate, accompanied by a sharp decrease in potential, which was attributed to the reduction in Cu²⁺ concentration caused by the formation of copper sulfide (Cu₂S), as indicated by arrow (c) in Figure 5. These results confirm the quick and reversible response of the fabricated electrode to variations in Cu²⁺ concentration resulting from the degradation of CuNPs.

### Quantification of copper ion contamination in environmental water samples from different sources

The fabricated Cu²⁺-selective electrode was applied to analyze copper ion content in different environmental water samples, including tap water, swimming pool water, Nile River water, and industrial wastewater, with distilled water used as a blank reference. The electrodes were immersed directly into each sample, and the corresponding electromotive force (EMF) was recorded.

In unspiked samples, Cu²⁺ was found to be below the detection limit (7.5 × 10⁻⁹ mol L⁻¹) in tap water, pool water, and Nile River water, indicating negligible copper contamination under the tested conditions. In contrast, industrial wastewater contained a measurable level of copper ions, confirming the presence of contamination likely due to industrial discharge. These findings are consistent with the well-documented impact of factory effluents on the Nile River and associated waterways [58]. Pool water is also known to sometimes contain added copper as an algaecide or to impart a blue color, with reported limits up to 0.2 mg/mL [59]; however, in the samples analyzed here, Cu²⁺ concentrations were not detectable.

To further validate the method, all water types were spiked with Cu²⁺ at a concentration of 1 × 10⁻⁴ mol L⁻¹. The developed electrode demonstrated excellent mean recovery values ranging from 95.8 to 98.9%, confirming its accuracy, reliability, and applicability in complex environmental matrices.

To further validate the analytical accuracy of the proposed potentiometric method, the obtained results were compared with those reported in the literature using established reference techniques such as atomic absorption spectrometry (AAS) and inductively coupled plasma optical emission spectrometry (ICP-OES). Han et al. (2023) determined ultra-trace copper in environmental water samples using dispersive liquid–liquid microextraction combined with graphite furnace AAS, achieving a detection limit of approximately 0.01 µg L⁻¹ and recoveries of 97–102% [60]. Although this laboratory-based technique offers greater sensitivity than the present potentiometric method (LOD = 0.48 µg L⁻¹), it requires complex pre-concentration and specialized instrumentation. In contrast, the current solid-contact Cu²⁺-selective electrode allows direct analysis without sample pretreatment, offering rapid and low-cost detection suitable for on-site monitoring.

In comparison, ICP-OES methods typically provide reliable multi-element quantification within the 1–10 µg L⁻¹ range. For example, Silva et al. reported a limit of quantification of 3.2 µg L⁻¹ for Cu in produced water and observed concentrations of several tens to hundreds of µg L⁻¹ [61], while Wu et al. demonstrated the applicability of ICP-OES for comprehensive water-quality monitoring programs [62]. The Cu²⁺ concentrations obtained in this study—below the detection limit (< 0.48 µg L⁻¹) for tap, pool, and Nile River water, and approximately 320 µg L⁻¹ in industrial wastewater, fall within the analytical range reported for these reference techniques and remain far below the WHO (2000 µg L⁻¹) and EPA (1300 µg L⁻¹) guideline values for copper in drinking water [63, 64]. These findings confirm that the proposed potentiometric sensor provides accuracy comparable to spectrometric methods while uniquely offering portability and real-time monitoring capabilities for both environmental and nanoparticle-synthesis applications.

## Greenness assessment of the method

In recent years, there has been an increasing emphasis on fostering a greener environment and ensuring a sustainable future [65, 66]. Consequently, it has become essential to develop new analytical methods that align with the principles of green chemistry [67]. To assess the environmental impact of analytical procedures more accurately and simply, several green metrics have been introduced. Among these, the analytical eco scale, GAPI, and Agree metrics were utilized to evaluate the greenness of the proposed copper ion-selective electrode method. The analytical eco scale assigns a score of 100 to the greenest analytical methods, with penalty points subtracted for certain aspects of the procedure. These aspects include the quantity and hazards associated with solvents, the energy consumption of the analytical instrument, the occupational hazards to the operator, and the total waste generated. Our proposed method achieved a score of 95, which qualifies it as an excellent green analytical method according to the Eco scale [68], as presented in Table 3.

**Table 3:** Analytical ECO scale greenness assessment

Parameter	Type used	Score
Amount of reagent	> 100 mL (g)	3
Hazard of solvent used	None (phosphate buffer)	0*3 = 0
Energy consumption	≤ 0.1 kWh per sample	0
Occupational hazard	Analytical process hermitization	0
Waste	> 10 mL (g)	5
Total penalty points:		5
Total score:		100-5 = 95 ∴ a green method

**Fig. 6:**
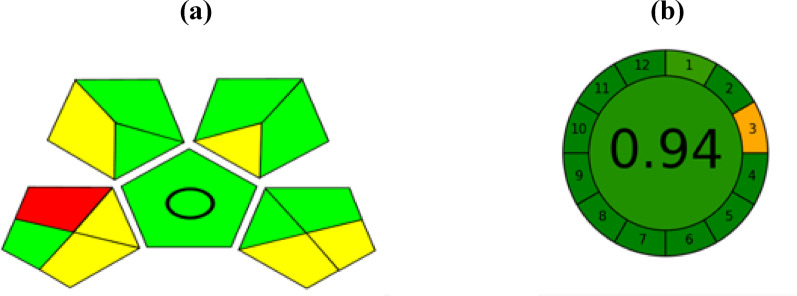
**a** GAPI generated pentagram for the proposed ion-selective electrode method. **b** AGREE software results of the proposed ion selective electrode method

The next metric used was the GAPI tool, which visually assesses the greenness of the method based on color coding, where green represents the most environmentally friendly, red indicates the least, and yellow is intermediate [69]. Only one compartment received the red color due to storing environmental samples at 4 °C. This step was applied as an extra precaution to avoid bacterial contamination. The green color was predominant in other compartments in the chart, highlighting the overall greenness of the method as shown in Fig. 6A. Finally, the Agree metric was used, which utilizes software to automatically calculate a final score based on the details of each step in the analytical procedure. Methods with scores greater than 0.8 are considered green methods, where the higher the scores approach 1, the greener the method [70]. The proposed method received a score of 0.94 with a dark green color indicating that it is environmentally friendly as shown in Fig. 6B. The results from all three metrics confirmed the greenness of the method, as it uses low-energy consuming instruments (a potentiometer) and an environmentally friendly solvent (phosphate buffer).

## Conclusion

A novel copper(II)-selective solid-contact ion-selective electrode was successfully developed and applied for real-time monitoring of copper ion dynamics during nanoparticle synthesis, degradation, and environmental water analysis. By integrating polyaniline nanoparticles as a stable ion-to-electron transducer and β-cyclodextrin as a selective ionophore, the sensor achieved a detection limit of 7.5 × 10⁻⁹ mol L⁻¹, rapid response time (≤ 10 s), and excellent stability within the pH range of 6–9.

For the first time, such an electrode was employed as an in situ kinetic and thermodynamic probe for copper nanoparticles formation and decay, enabling calculation of activation energy, enthalpy, and Gibbs free energy directly from potentiometric measurements during copper nanoparticle formation and oxidative degradation. This represents a significant advance, as these parameters are traditionally accessible only through more complex and expensive instrumentation.

Application to real water samples further demonstrated the electrode’s utility. Copper was detected in industrial wastewater, while tap, pool, and Nile River waters showed concentrations below the detection limit. Spiking experiments yielded recoveries between 95.8 and 98.9%, confirming the method’s reliability and robustness in diverse environmental matrices.

The developed sensor provides a green, low-cost, and portable platform for copper monitoring, aligning with the principles of sustainable analytical chemistry. Its dual capability as both a nanoparticle process-monitoring tool and an environmental sensor highlights its potential for broad application in nanotechnology, environmental quality assessment, and future point-of-care testing.

## Supplementary Information

Supplementary Material 1: Figure 1s: Effect of pH changes on the response of copper ion selective electrode using solutions of BRB buffers with different pH values. The results represent the average of three determinations. Figure 2s: The potentiometric aqueous layer test. Figure 3s: Determination of the rate for reduction at different temperatures. Figure 4s: Arrhenius plot of ln*k *against 1/T. Eyring plot of lnK/T against 1/T.

## Data Availability

All data generated or analyzed during this study are included in this article and the raw data is available from the corresponding author if requested.
