# Supplementary material for: A nanostructured solid-contact electrode for real-time monitoring of copper nanoparticle dynamics and environmental analysis
Source: BMC Chem. 2025 Dec 16;20(1):14. doi: 10.1186/s13065-025-01692-y (PMC12822006; doi:10.1186/s13065-025-01692-y)
Supplement: Supplementary file 1 — Supplementary Material 1: Figure 1s: Effect of pH changes on the response of copper ion selective electrode using solutions of BRB buffers with different pH values. The results represent the average of three determinations. Figure 2s: The potentiometric aqueous layer test. Figure 3s: Determination of the rate for reduction at different temperatures. Figure 4s: Arrhenius plot of lnk against 1/T. Eyring plot of lnK/T against 1/T. [file 13065_2025_1692_MOESM1_ESM.docx]

**Supplementary files**

**Figure (1s):** Effect of pH changes on the response of copper ion selective electrode using solutions of BRB buffers with different pH values. The results represent the average of three determinations.

**Figure (2s):** The potentiometric aqueous layer test.

**Figure (3s):** Determination of the rate for reduction (K) at different temperatures

**Figure (4s):** (A) Arrhenius plot of $\ln k$ against 1/T. (B) Eyring plot of $\ln\frac{K}{T}$ against 1/T.
